# Neutrophil-to-lymphocyte Ratio and Platelet-to-lymphocyte Ratio in Patients with Retinal Artery Occlusion

**DOI:** 10.18502/jovr.v15i2.6737

**Published:** 2020-04-06

**Authors:** Mahmut Atum, Gürsoy Alagöz

**Affiliations:** Department of Ophthalmology, Sakarya University Education and Research Hospital, Adnan Menderes Caddesi Saglik Sokak, Sakarya, Turkey

**Keywords:** Hemogram, Neutrophil-to-Lymphocyte Ratio, Platelet-to-Lymphocyte Ratio, Retinal Artery Occlusion, Retinal Vessels

## Abstract

**Purpose:**

This study aimed to compare the neutrophil-to-lymphocyte (NLR) and platelet-to-lymphocyte (PLR) ratios in patients with retinal artery occlusion (RAO) with those from a healthy control population and to identify the relationship between them.

**Methods:**

Forty-six patients with RAO and fifty-one healthy control subjects were included in this retrospective case-control study. RAO was diagnosed following an ophthalmic examination and fluorescein angiography (FA). Blood neutrophil, lymphocyte, and platelet counts were recorded for each of the 97 subjects, from which NLR and PLR values were calculated.

**Results:**

There were 46 patients (28 male [M], 18 female [F]) in the RAO group and 51 patients (27 M, 24 F) in the control group. No significant differences were found between patients with RAO and the control subjects in terms of gender and age (P > 0.05). Patients with RAO had significantly increased NLR values (2.85 ± 1.70) than the control subjects (1.63 ± 0.59, *P* < 0.001). The mean PLR in patients with RAO was 123.69 ± 64.98, while that in control subjects was 103.08 ± 36.95; there was no significant difference between the two groups (*P* = 0.055). A logistic regression analysis revealed that NLRs were 3.8 times higher in patients with RAO than in control subjects (odds ratio = 3.880; 95% confidence interval = 1.94 to 7.74; *P* < 0.001).

**Conclusion:**

NLRs were significantly increased in patients with RAO compared to the control subjects.

##  INTRODUCTION

Retinal artery occlusion (RAO), generally seen in older adults,^[1]^ is a serious condition requiring emergency intervention. RAO is characterized by sudden unilateral vision loss and/or visual field defect.^[2]^ There are three types of RAO: central retinal artery occlusion (CRAO), branch retinal artery occlusion (BRAO), and cilioretinal artery occlusion (CLRAO).^[3]^ The distribution of the RAO sub-types is as follows: CRAO, 57%; BRAO, 38%; and CLRAO 5%.^[3]^

RAOs are usually caused by embolisms, which in turn are often caused by atherosclerotic plaques associated with carotid artery disease.^[4]^ Arruga et al showed that emboli causing RAO are composed of cholesterol material (74%), calcific material (10.5%), and/or fibrin material (15.5%).^[5]^ Atherosclerosis is a chronic inflammatory condition,^[6]^ and thrombosis and inflammation have been shown to be interconnected in a complex manner.^[7]^

Several previous studies have shown that a patient's neutrophil-to-lymphocyte ratio (NLR) and platelet-to-lymphocyte ratio (PLR) are indicative of systemic inflammation.^[8,9,10,11]^ Mean platelet volume (MPV) is also indicative of the status of platelets and is also associated with inflammation.^[12]^ In their literature review, Şahin et al found that only one study analyzed the relationship between MPV and RAO showing that MPV values were significantly higher in patients with RAO.^[13]^ We could not find any publication that examined the association between NLR, PLR, and RAO. In this study, we compared the NLR and PLR levels in patients with RAO with those of healthy control subjects and examined the relationship between these values.

##  METHODS 

This retrospective study was performed in the Sakarya Training and Research Hospital Eye Disease Polyclinic. The records of patients diagnosed with CRAO, BRAO, and CLRAO between January 2016 and September 2018 were analyzed. The study included 46 patients with RAO and 51 healthy control subjects. The RAO patient group comprised of individuals who experienced sudden, painless loss of vision and were subsequently diagnosed with RAO in an ophthalmology outpatient clinic. The control group consisted of subjects who presented with impaired vision and underwent cataract surgery. A routine blood analysis was performed before any cataract surgery in our clinic. Age and sex were similar in the two groups, as was the number of patients with systemic hypertension.

All subjects underwent a complete ophthalmic evaluation in both eyes, including a test of visual acuity using a Snellen chart, inspection of the anterior and posterior segments with a slit-lamp biomicroscope, and applanation tonometry. Fluorescein angiography (FA) was performed in all patients, and the diagnosis of RAO was defined accordingly.

The exclusion criteria for this study were as follows: cardiovascular diseases (such as congestive heart failure and heart valve disease treated with an anticoagulant), diabetes, history of stroke, history of smoking, blood disorders, anemia, renal failure, hepatic disorders, malignancies, and vasculitis. Patients with a history of eye surgery, glaucoma, or eye trauma were also excluded.

Blood samples were taken from each RAO patient within 2 h of diagnosis. The hemogram parameters of each subject were measured using a Cell-DYN 3700 automated hematology analyzer (Abbott Diagnostics, Abbott Park, IL, USA). The hemogram (neutrophil, lymphocyte, and platelet) results were entered in an Excel (Microsoft Corp., Redmond, WA, USA) spreadsheet to calculate their NLRs and PLRs.

This study was conducted according to the principles of the Declaration of Helsinki and the approval was obtained from the institutional ethics committee. Ethical approval was obtained from the local Ethics Committee.

### Statistical Analysis

Data were analyzed using SPSS software version 18.0 (SPSS Inc., Chicago, IL, USA). The results of numerical data analysis were given as mean and standard deviation. The independent groups were compared using a parametric Student's *t-*test. The cut-off point for NLR and PLR between patients with RAO and control subjects was determined using a receiver operating characteristic (ROC) curve analysis. Sensitivity and specificity were determined according to cut-off values. A univariate logistic regression analysis was used to determine the association between RAO and NLR, PLR, age, and sex data. The results were evaluated at a 95% confidence interval (CI) and *P* < 0.05 was considered significant.

##  RESULTS

This study consisted of 97 patients: 46 patients with RAO and 51 healthy control subjects. The RAO group consisted of 28 men and 18 women and the control group included 27 men and 24 women. The mean age of patients with RAO was 63.02 ± 14.80 years and that of the control subjects was 61.33 ± 7.91 years. No significant difference was found between the RAO and control subjects in terms of the gender (*P* = 0.437) or age (*P* = 0.479) (all data are summarized in Table 1).

**Table 1:** Demographic features and laboratory findings in the RAO and control groups


	**RAO (** ***n*** ** = 46)**	**CONTROL (** ***n*** ** = 51)**	***P*** **-value**
Age (y)	63.02 ± 14.80	61.33 ± 7.91	0.479
Sex (M/F)	28/18	27/24	0.437
WBC (10 ${}^{9}$ /L)	7.90 ± 2.16	7.48 ± 2.34	0.366
Neutrophil (10 ${}^{9}$ /L)	5.05 ± 1.88	4.00 ± 1.51	**0.003**
Lymphocyte (10 ${}^{9}$ /L)	2.05 ± 0.75	2.64 ± 1.12	**0.003**
Platelet (10 ${}^{9}$ /L)	222.96 ± 67.72	242.60 ± 52.91	0.113
NLR	2.85 ± 1.70	1.63 ± 0.59	**<0.001**
PLR	123.69 ± 64.98	103.08 ± 36.95	0.055
MPV (fL)	8.27 ± 1.04	8.49 ± 1.88	0.652
Bold values are statistically significant measurements (Independent samples *t*-test) F, female; M, male; MPV, mean platelet volume; NLR, neutrophil-to-lymphocyte ratio; PLR, platelet-to-lymphocyte ratio; RAO, retinal artery occlusion; WBC, white blood cell			

The mean white blood cell count was 7.90 ± 2.16 10 ${}^{9}$ /L in the RAO group and 7.48 ± 2.34 10 ${}^{9}$ /L in the control group; no significant difference was found between the two groups (*P* = 0.366). The mean neutrophil count of the RAO group was 5.05 ± 1.88 10 ${}^{9}$ /L, which was significantly higher than that of the control group (4.00 ± 1.51 10 ${}^{9}$ /L) (*P* = 0.003). The mean lymphocyte count was 2.05 ± 0.75 10 ${}^{9}$ /L for the RAO group and 2.64 ± 1.12 10 ${}^{9}$ /L for the control group, indicating a significantly lower value in patients with RAO (*P* = 0.003). The mean platelet count was 222.96 ± 67.72 10 ${}^{9}$ /L in the RAO group and 242.60 ± 52.91 10 ${}^{9}$ /L in the control group, showing no significant difference between the two groups (*P* = 0.113).

The mean NLR of patients with RAO was 2.85 ± 1.70, while that of the control subjects was 1.63 ± 0.59, revealing that NLR levels were significantly higher in the RAO group (*P* < 0.001). The mean PLR was 123.69 ± 64.98 in patients with RAO and 103.08 ± 36.95 in control subjects, indicating that there was a borderline level of statistical significance between the two groups (*P* = 0.055) [Table 1]. Finally, the mean MPV values were 8.27 ± 1.04 fL for the RAO group and 8.49 ± 1.88 fL for the control group; no significant difference was found between the two groups (*P* = 0.652).

**Figure 1:**
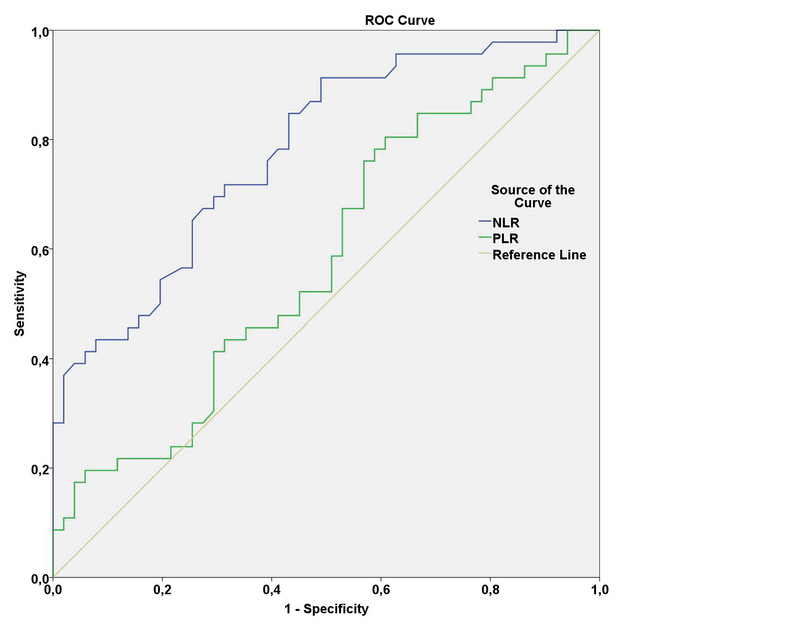
ROC curve analysis of NLR and PLR in RAO patients. NLR was determined to be more sensitive and had higher rate as a predictor of inflammation compared to PLR. AUC for NLR: 0.780, cut-off value: 1.82, sensitivity: 72%, specificity: 69%. (95% CI: 0.690–0.871). AUC for PLR: 0.582, cut-off value: 92.35, sensitivity: 67%, specificity: 57%. (95% CI: 0.468–0.696). AUC, area under the ROC curve; NLR: neutrophil-to-lymphocyte ratio; PLR platelet-to-lymphocyte ratio; RAO: retinal artery occlusion.

A ROC curve is a graphical plot that illustrates the diagnostic ability. ROC analysis was performed for the NLR values, and the area under the curve, cut-off value, sensitivity, and specificity were 0.780, 1.82, 72%, and 69%, respectively (95% CI: 0.690–0.871). For the PLR values, the area under the curve, cut-off value, sensitivity, and specificity were 0.582, 92.35, 67%, and 57%, respectively (95% CI: 0.468–0.696) [Figure 1]. According to the logistic regression analysis we performed, NLR was in fact an indicator for RAO (Odds ratio (OR) = 3.880; 95% CI = 1.94–7.74; *P* < 0.001). But PLR was not an independent indicator of RAO. (OR = 1.036; 95% CI = 0.999-1.012; p = 0.126).

##  DISCUSSION

In this study, we found that NLR levels were significantly increased in patients with RAO, demonstrating that it is in fact an independent indicator of RAO. Patients with high NLR levels are 3.8 times more likely to have RAO than patients with low NLR levels. This study is the first to examine the association between NLR and RAO.

Previous studies have shown that embolisms are the most common cause of RAO, and that the main cause of embolisms is carotid artery disease caused by atherosclerosis.^[4]^ Atherosclerosis is associated with chronic inflammation,^[6,14]^ so it is possible that inflammatory biomarkers may play a significant role in predicting which patients may develop RAO.

NLR, which is calculated based on dividing a patient's number of neutrophils by the lymphocyte count, is a simple and cheap indicator of systemic inflammation. Systemic inflammation typically involves lymphopenia and neutrophilia.^[15]^ In their retrospective study, Gokhan et al reported that NLR was found to be an independent marker in cases of symptomatic carotid artery disease; it was also found to be higher in symptomatic than asymptomatic patients suffering from a stroke or a transient ischemic attack (*P* < 0.001).^[16]^ In a different study, Tokgoz et al showed that NLR is an important indicator of prognosis and mortality in stroke patients.^[17]^ A meta-analysis showed that NLR may play a major role in the diagnosis and prognosis of peripheral vascular diseases.^[18]^ In the current study, NLR was revealed to be significantly increased in patients with RAO compared to control subjects, which may be further evidence that chronic inflammation may lead to the development of RAO. Logistic regression analysis indicated that NLR is an independent indicator of RAO.

Dursun et al compared 40 patients with retinal vein occlusion with a control group and found that the NLR levels of patients with retinal vein occlusion were significantly higher than those in control patients.^[19]^ The sensitivity and specificity of NLR reported by Dursun et al were found to be 72% and 100%, respectively (cut-off value: 1.89), whereas in our study, these figures were 72% and 69%, respectively (cut-off value: 1.82).^[19]^

PLR, which is calculated via dividing a patient's platelet count by the number of lymphocytes, is a cheap and easy test that reveals the condition of platelets and white blood cells. Thrombocytes play a significant role in coronary artery disease and cardiovascular disorders.^[20]^ Research has also shown that platelets play a significant role in the development of atherosclerosis and embolisms.^[21]^ Azab et al identified a relationship between increased PLR and long-term mortality in patients with myocardial infarction,^[11]^ while Ferroni et al reported a relationship between increased PLR and the risk of venous thromboembolism.^[22]^ In their review, Balta et al demonstrated that a high PLR level is associated with inflammation, atherosclerosis, and activated platelets.^[23]^ Our study showed that patients with RAO had higher PLRs than control subjects; however, logistic regression analysis showed that PLR cannot be used as an indicator of RAO.

Şahin et al reported that MPV values were significantly higher in patients with RAO than in control subjects,^[13]^ but we were unable to replicate this finding. We found no significant difference between the two groups in the current study. We believe that this discrepancy may be due to the difference in the number of patients in the respective studies.

Many cardiovascular disorders decrease the lymphocyte counts. Bian et al reported that decreased lymphocyte levels can be used as a marker of acute coronary syndrome,^[24]^ and several other studies have shown that a reduced percentage of lymphocytes in patients with acute heart failure is associated with morbidity and mortality.^[25,26]^ However, Cooper et al reported that an increased neutrophil count could also be a marker of mortality in cases with left ventricular dysfunction.^[27]^ Similar to these studies, we found that blood neutrophil levels were significantly increased and lymphocyte counts were significantly decreased in patients with RAO than in control subjects.

This study had a few limitations. We examined a small number of patients using a retrospective design and did not assess body mass index and associated atherosclerosis. Further studies including a larger number of patients are necessary to better investigate the role of serum NLR and PLR in RAO disease.

In conclusion, we found that NLRs were significantly higher and lymphocyte counts were significantly lower in patients with RAO than in control subjects. Larger studies are needed to better understand the relationship between RAO and NLR.
